# Parameter Identification of the Fractional-Order Mathematical Model for Convective Mass Transfer in a Porous Medium

**DOI:** 10.3390/membranes13100819

**Published:** 2023-09-28

**Authors:** Ivan Pavlenko, Marek Ochowiak, Sylwia Włodarczak, Andżelika Krupińska, Magdalena Matuszak

**Affiliations:** 1 Department of Computational Mechanics named after Volodymyr Martsynkovskyy, Sumy State University, 2, Rymskogo-Korsakova St., 40007 Sumy, Ukraine; 2 Department of Chemical Engineering and Equipment, Poznan University of Technology, 5, M. Skłodowskiej-Curie Sq., 60-965 Poznan, Poland; marek.ochowiak@put.poznan.pl (M.O.); sylwia.wlodarczak@put.poznan.pl (S.W.); andzelika.krupinska@put.poznan.pl (A.K.); magdalena.matuszak@put.poznan.pl (M.M.)

**Keywords:** advanced process, Riemann–Liouville fractional derivative, Mittag-Leffler function, process innovation

## Abstract

Fractional calculus is an essential tool in studying new phenomena in hydromechanics and heat and mass transfer, particularly anomalous hydromechanical advection–dispersion considering the fractal nature of the porous medium. They are valuable in solving the urgent problem of convective mass transfer in a porous medium (e.g., membranes, filters, nozzles, convective coolers, vibrational prillers, and so on). Its solution allows for improving chemical engineering and technology workflows, refining process models for obtaining porous granular materials, realizing the convective cooling of granular and grain materials, and ensuring the corresponding apparatuses’ environmental safety. The article aims to develop a reliable convective mass transfer model for a porous medium and proposes a practical approach for its parameter identification. As a result, a general scientific and methodological approach to parameter identification of the fractional convective mass transfer model in a porous medium was proposed based on available experimental data. It mainly used Riemann–Liouville fractional time and coordinate derivatives. The comprehensive application of the Laplace obtained the corresponding general solution transform with respect to time and a coordinate, the Mittag-Leffler function, and specialized functions. Different partial solutions in various application case studies proved this solution. Moreover, the algorithm for practically implementing the developed approach was proposed to evaluate parameters for the considered model by evaluation data. It was reduced to the two-parameter model and justified by the available experimental data.

## 1. Introduction

Research on the transport phenomena across porous membranes became a significant problem many years ago [1]. This process was accompanied by the development of membrane science [2]. Successful attempts to advance solving the problem of convective mass transfer in porous membranes have gained more importance recently [3]. Differential equations that describe the convective mass transfer process present significant difficulties for their solution [4]. An analytical solution to these equations can be practically expedient only for cases with significant simplifications [5].

Therefore, approaches based on the similarity theory are frequently used to solve this problem [6]. However, using algebraic criterion equations significantly limits understanding of the physical meaning of mass transfer processes [7].

Moreover, using porous materials predetermines the need to consider the fractional derivatives in mass transfer equations [8]. Standard partial differential equations cannot study relevant phenomena occurring in such media [9].

In this regard, the problem of considering fractional-order partial differential equations and their subsequent analytical solution for specific boundary and initial conditions is a fundamental scientific problem. Moreover, the practical significance of solving the parameter identification problem of the fractional convective mass transfer model in a porous medium is highlighted by the need to intensify heat and mass transfer processes in energy-efficient apparatuses of the chemical industry (e.g., convective coolers for granular and grain materials, vibrational prilling of fertilizers, and so on).

Numerous key publications in the related fields also highlight the significance of this problem. Li and Kurths [10] proposed a numerical algorithm for solving the fractional convective diffusion equation. As a result, the behavior of the fractional convection model was analyzed. Salomoni and De Marchi [11] described a nonlinear anomalous hydromechanical advection–dispersion model. As a result, fractional advection–diffusion equations using the Grünwald–Letnikow definition of a fractional derivative were solved numerically.

Allwright and Atangana [12] considered the fractional-order advection–dispersion equation for groundwater transport. As a result, the fractional integral of a system with self-similarities was calculated numerically. Anwar et al. [13] investigated heat transfer in a nanofluid flow through a porous medium. As a result, the friction coefficient and Nusselt number were calculated numerically for the fractional derivative model.

Madhura et al. [14] studied the impact of nanoparticle shapes on natural convection flow with heat and mass transfer rates of nanofluids with fractional derivatives. As a result, the governing fractional-order differential equations were solved using the Laplace transform with boundary conditions. However, this model does not consider fractional derivatives for porous media.

Borah et al. [15] numerically analyzed thermal transfer equations for non-Newtonian fluids. Based on Atangana–Baleanu and Caputo–Fabrizio fractional derivatives, the mixed convective unsteady flow through an infinite vertical porous plate was investigated for a uniform field. Li et al. [16] applied a fractional lattice Boltzmann method to solve multiphase flows with complex interfacial behavior and significant density contrast. As a result, a good agreement was obtained between the numerical results and the available numerical data.

Raza et al. [17] proposed a non-singular fractional approach for the thermal analysis of natural convection in nanofluids. As a result, an impact of the fractional parameter on temperature and velocity fields was obtained graphically. Khan et al. [18] considered free convection flow between two parallel plates. As a result, the coupled system of Fick’s and Fourier’s equations was solved numerically using Python 3.11.5 software.

Rehman et al. [19] proposed generalized Mittag-Leffler solutions of free convection heat and mass transfer flow of the Maxwell fluid with Newtonian heating. As a result of using the Prabhakar fractional derivative approach, a comparison between velocity and temperature fields for Maxwell and Newtonian fluids for both fractional and classical cases with and without slip conditions was provided. A similar problem was solved semi-analytically by Riaz et al. [20].

Ali et al. [21] solved a generalized magnetohydrodynamic two-phase free convection flow model of dusty Casson fluid between parallel plates. As a result, the temperature field was plotted using the MathCAD 14 software. However, these studies did not consider the fractional derivatives with respect to time coordinates in the governing equations. Meng et al. [22] numerically solved the fractional viscoelastic fluid problem. As a result of using the finite difference approach, the complexity of solving the coupled system of temperature and concentration equations was highlighted. However, fractional convection was not considered.

Convection in a porous medium is influenced by a significant number of factors (hydrodynamic, temperature, and so on). Their comprehensive impact on the features of the operating processes can be initially determined by the criteria of similarity (e.g., Reynolds, Nusselt, Prandtl) given in the work [23]. However, the integer-order derivatives describing the acting forces in the process do not allow for considering either the phenomenon of anomalous convection at low Prandtl numbers described in the work [24] or the fractal nature of the medium [25]. Therefore, the fractional-order mathematical model should be considered, and the parameter identification approach by available experimental results data should be developed.

Due to the abovementioned analysis, the following research gaps should be eliminated despite the numerous studies in this field. Firstly, preconditions for possible analytical solutions of the fractional convective mass transfer equation should be considered. The available partial solutions should justify these solutions for a continuous medium and integer time dimension. Secondly, a scientific approach for parameter identification of the stationary model should be applied. The corresponding methodology for evaluating the fractional order by experimental data of concentration changes should be proposed. It should also be extended for the case of the non-stationary model.

Therefore, the main aim of the research is to develop a reliable convective mass transfer model for a porous medium and propose an effective approach for its parameter identification based on available experimental data.

## 2. Materials and Methods

### 2.1. A Flowchart of the Research

**Figure 1:**
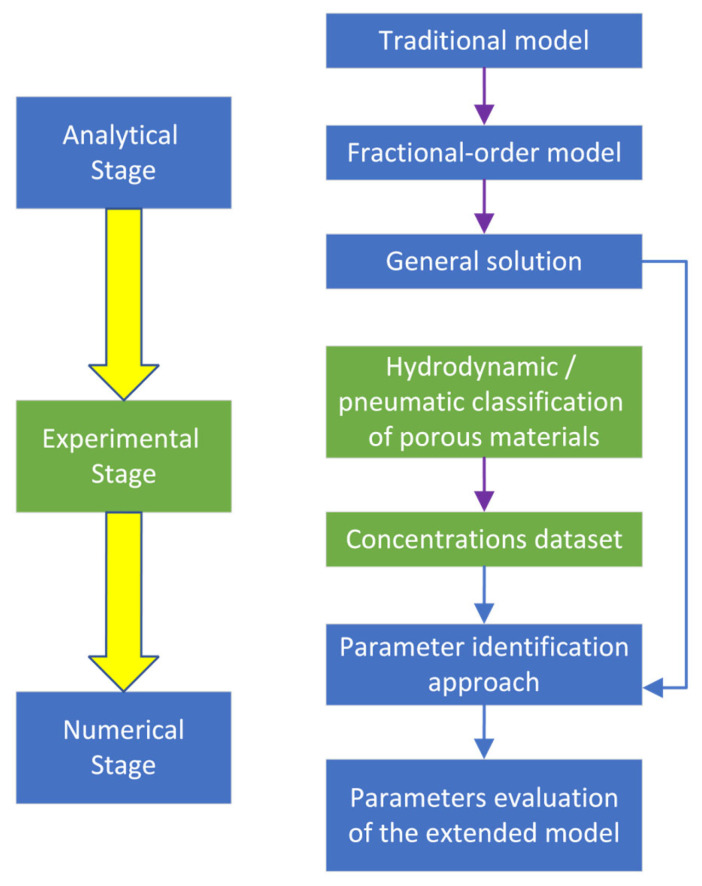
The flowchart of the research.

A flowchart of the research methodology is presented in Figure 1. It consists of the following three main stages: analytical (both classical and a proposed new one), experimental (obtaining results in a hydrodynamic apparatus), and numerical (evaluating the parameters of the fractional order mathematical model).

The first (analytical) stage allows for identifying the problems with the traditional model (e.g., inability to describe certain hydrodynamic phenomena) and observing ways for its further extension.

The second (experimental) stage allows for obtaining experimental results data for the practical case study (e.g., pneumatic classification of porous material in a rhomb-shaped hydrodynamic apparatus).

The last (numerical) stage allows for approximating the evaluated results data to make fundamentals of the parameter identification of the proposed fractional-order mathematical model. It should also justify the reliability of this model in terms of its compliance with available data.

### 2.2. The Traditional Model

The general convection–diffusion equation in a vector form is as follows [26]:

(1) $$\frac{\partial c(\vec{r},t)}{\partial t}=-\nabla \cdot [{\vec{U}}_{0}(\vec{r},t)\cdot c(\vec{r},t)]+\nabla \cdot [D_{0}(\vec{r},t)\cdot \nabla c(\vec{r},t)]+R(\vec{r},t),$$

where $c(\vec{r},t)$—mass concentration field in a vector form, kg/kg; $\vec{r}$—radius-vector, m; *t*—time, s; ${\vec{U}}_{0}(\vec{r},t)$—vector velocity field, m/s; $D_{0}(\vec{r},t)$—diffusion coefficient, m^2^/s; $R(\vec{r},t)$—current source, kg/s.

Despite the complexity of this spatial-dimension model, the following one-dimensional model with unchanged velocity *U*_0_ and diffusion coefficient *D*_0_ is practically applied in studying problems of filtration through porous medium [27], the energy efficiency of liquid–vapor ejectors [28], inertial-filtering separation of gas–liquid mixtures [29], and pneumatic classification of granular material [30]:

(2) $$\frac{\partial c\left(z,t\right)}{\partial t}=-U_{0}\frac{\partial c\left(z,t\right)}{\partial z}+D_{0}\frac{\partial^{2}c\left(z,t\right)}{\partial z^{2}}+R\left(z,t\right),$$

where *z*—coordinate, m; *c*(*z*, *t*)—mass concentration, kg/kg; *R*(*z*, *t*)—specific driving force, kg/s.

However, even this equation does not consider the fractional origin of the process in a porous medium. Therefore, it should be rewritten considering Riemann–Liouville fractional derivatives with respect to time and dimension for a porous medium despite standard integer dimensions.

The initial equations contain the integer-order partial differential equations. However, these models do not allow for considering either the anomalous convection phenomenon or the medium’s fractal structure. This is because such phenomena can be studied by considering the fractional-order derivatives. Therefore, these equations should be extended to a general fractional-order mathematical model using fractional derivatives. Moreover, a scientific approach should be developed to identify the model’s parameters by available experimental results data.

### 2.3. The Considered Fractional-Order Convection Model

For the reasons mentioned above, the following assumptions and hypotheses are proposed. Firstly, the process is predominantly convective rather than diffusive for significant values of the comprehensive criterion *Re*·*Pr*, where Re—Reynolds number; Pr—Prandtl number. In this case, the diffusion component can be neglected compared to the convective one.

Secondly, for a fractional convective process, the time derivative of the mass concentration should be rewritten as a fractional derivative $\frac{\partial^{\alpha }c\left(z,t\right)}{\partial t^{\alpha }}$, where *α* = (0, 1]—the fractional order (for the standard integer order *α* = 1). Additionally, for a porous medium, the convective mass transfer component should be rewritten as $-U_{0}\frac{\partial^{\beta }c\left(z,t\right)}{\partial z^{\beta }}$, where *β* = (0, 1]—the fractional order (for a continuous medium *β* = 1); $U_{0}$—convective ratio as a fractional analog of the carrier flow velocity. In this case, the following Riemann–Liouville fractional derivatives [31,32] are considered:

(3) $$\frac{\partial^{\alpha }c\left(z,t\right)}{\partial t^{\alpha }}=\frac{1}{\Gamma \left(1-\alpha \right)\;}\frac{\partial }{\partial t}\int_{0}^{t}\frac{c\left(z,\tau \right)}{{\left(t-\tau \right)}^{\alpha }}d\tau ;\;\;\frac{\partial^{\beta }c\left(z,t\right)}{\partial z^{\beta }}=\frac{1}{\Gamma \left(1-\beta \right)\;}\frac{\partial }{\partial z}\int_{0}^{z}\frac{c\left(\zeta ,t\right)}{{\left(z-\zeta \right)}^{\beta }}d\zeta ,$$

where Γ(*θ*)—Euler’s gamma function of the argument *θ*; *τ*, *ζ*—independent time and coordinate parameters, respectively.

Fourthly, the arbitrary specific driving force of the mass transfer process can be presented in a linearized form as *R*(*z*, *t*) = *K*·[*c_s_* − *c*(*z*, *t*)], where *K*—mass transfer coefficient, kg/s; *c_s_*—limiting mass concentration of the dispersed phase under the saturation, kg/kg. In this case, a constant value of the mass transfer coefficient *K* is usually considered for the interfacial surface.

Moreover, since the convective mass transfer process occurs due to the change in limiting *s_s_* and current *c* concentrations and simplifying the below-mentioned transformations, zero boundary and initial conditions can be used: *c*(0, *t*) = *c*(*z*, 0) = 0. Furthermore, these conditions will, in the future, allow us not to complicate the problem with the traditional procedure of eliminating singularities associated with the application of Riemann–Liouville derivatives.

Finally, the stationary mode occurs for significant values of the comprehensive criterion $\frac{Fo\cdot Pr}{Re}$, where Fo—diffusion Fourier number. This result entirely corresponds to the stationary convection equation describing the steady-state behavior of such systems. In this case, the time derivative can be neglected, and the concentration field leads to the limiting function $c_{\infty }\left(z\right)$ with the analogous boundary condition $c_{\infty }\left(0\right)=0$.

Under the assumptions mentioned above, the following differential equations as the fractional convective mass transfer models in a porous medium are considered:

(4) $$\frac{\partial^{\alpha }c\left(z,t\right)}{\partial t^{\alpha }}=-U_{0}\frac{\partial^{\beta }c\left(z,t\right)}{\partial z^{\beta }}+K\left[c_{s}-c\left(z,t\right)\right];$$

(5) $$0=-U_{0}\frac{\partial^{\beta }c_{\infty }\left(z\right)}{\partial z^{\beta }}+K\left[c_{s}-c_{\infty }\left(z\right)\right].$$

These equations should be solved analytically for the above boundary and initial conditions.

### 2.4. Analytical Solution of the Non-Stationary Equation

The consequent application of the Laplace transform is used to solve fractional convective mass transfer Equations (4) and (5).

Equation (4), considering the mentioned boundary and initial conditions, takes the following form:

(6) $$\tau^{\alpha }C\left(s,\tau \right)=-U_{0}s^{\beta }C\left(s,\tau \right)+K\left[\frac{c_{s}}{s\tau }-C\left(s,\tau \right)\right],$$

where *s*, *τ*—derivative operators with respect to longitudinal direction and time, respectively; *C*(*s*, *τ*) = ℒ*_t_*{ℒ*_s_*[*c*(*z*, *t*)]}—Laplace transform with respect to both coordinate and time.

The following result can be derived from the last equation:

(7) $$C\left(s,\tau \right)=\frac{Kc_{s}}{s\tau \left(Us^{\beta }+\tau^{\alpha }+K\right)}=c_{s}\left(\frac{1}{\tau }\cdot \frac{1}{s}-\frac{1}{\tau }\cdot \frac{s^{\beta -1}}{s^{\beta }+\frac{K}{U_{0}}}-\frac{1}{s}\cdot \frac{\tau^{\alpha -1}}{\tau^{\alpha }+U_{0}s^{\beta }+K}+\frac{\tau^{\alpha -1}}{\tau^{\alpha }+U_{0}s^{\beta }+K}\cdot \frac{s^{\beta -1}}{s^{\beta }+\frac{K}{U_{0}}}\right).$$

After the inverse Laplace transform with respect to both coordinate and time, the following general solution $c\left(z,t\right)=L_{s}^{-1}\left\{L_{\tau }^{-1}C\left(s,\tau \right)\right\}$ can be obtained:

(8) $$c\left(z,t\right)=c_{s}\left\{1-E_{\beta ,1}\left(-\frac{K}{U_{0}}z\right)+L_{s}^{-1}\left[\frac{s^{\beta -1}}{s^{\beta }+\frac{K}{U_{0}}}E_{\alpha ,1}\left[-\left(U_{0}s^{\beta }+K\right)t\right]\right]-L_{s}^{-1}\left[\frac{1}{s}E_{\alpha ,1}\left[-\left(U_{0}s^{\beta }+K\right)t\right]\right]\right\},$$

where *E_α_*_,1_, *E_β_*_,1_—Mittag-Leffler functions:

(9) $$E_{\alpha ,1}\left(-Kt\right)=\sum_{i=0}^{\infty }\frac{{\left(-Kt\right)}^{i}}{\Gamma \left(\alpha i+1\right)};E_{\beta ,1}\left(-\frac{K}{U_{0}}z\right)=\sum_{i=0}^{\infty }\frac{{\left(-\frac{K}{U_{0}}z\right)}^{i}}{\Gamma \left(\beta i+1\right)}$$

where $\Gamma \left(\beta i+1\right)=\int_{0}^{\infty }\theta^{\beta i}e^{-\theta }d\theta$—Euler’s gamma function; *i* = [0, ∞)—integer number; *θ*—independent variable.

### 2.5. Justification of the Obtained Solutions

Due to the complexity of the obtained general solution, its reliability should be proven for the following four reduced case studies. The first considers the traditional approach when *α* and *β* are integer numbers, and the second is for its stationary mode. The last two justifications should be based on reducing the general solution (8) to the stationary mode and the case without significant convection.

In justification of the reliability of the obtained solutions, Equation (8) should be a reduction to the traditional approach when *α* = *β* = 1. In this case, the following solution can be obtained:

(10) $$c\left(z,t\right)=c_{s}\left\{1-\left[1-H\left(t-\frac{z}{U_{0}}\right)\right]e^{-Kt}-e^{-\frac{K}{U_{0}}z}H\left(t-\frac{z}{U_{0}}\right)\right\},$$

where *H*(*t*)—Heaviside function.

**Figure 2:**
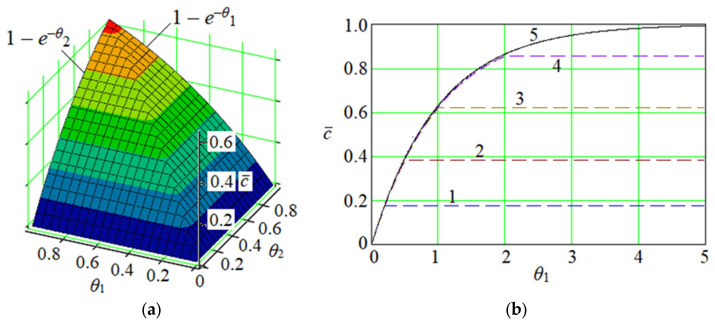
The reduced dimensional solution (11): (**a**)—the spatial surface; (**b**)—plane curves for different values of *θ*_2_: 1—0.2; 2—0.5; 3—1.0; 4—2; 5—∞.

This solution can be represented graphically in the following dimensionless form (Figure 2) for the case of K≥0 and $U_{0}\geq 0$:

(11) $$\bar{c}\left(\theta_{1},\theta_{2}\right)=1-e^{-\theta_{2}}\left[1-\left(1-e^{\theta_{2}-\theta_{1}}\right)H\left(\theta_{2}-\theta_{1}\right)\right],$$

where $\theta_{1}=Kt$—dimensionless time; $\theta_{2}=\frac{K}{U_{0}}z$—dimensionless longitudinal coordinate; $\bar{c}=\frac{c}{c_{s}}$—dimensionless concentration.

Notably, the reduced solution (10) entirely corresponds with the available solution [33] of Equation (2). Therefore, the obtained general solution of the fractional convective mass transfer model in a porous medium is valid.

Moreover, for the stationary mode ($t\geq \frac{z}{U_{0}}$; $c=c_{\infty }\left(z\right)=invar\left(t\right)$), the last equation takes the following form:

(12) $$c_{\infty }\left(z\right)=c_{s}\left(1-e^{-\frac{K}{U_{0}}z}\right)$$

that corresponds to the saturation equation [34]. This fact also proves the obtained general solution (8).

For the stationary mode, when *t* → ∞, two last inverse Laplace transforms in Equation (8) are reduced to 0, and concentration *c*(*z*, *t*) is reduced to its limiting function $c_{\infty }\left(z\right)=\underset{t\to \infty }{\lim}c\left(z,t\right)$. Therefore, the following solution for the stationary mode is obtained:

(13) $$c_{\infty }\left(z\right)=c_{s}\left[1-E_{\beta ,1}\left(-\frac{K}{U_{0}}z\right)\right].$$

This solution can also be obtained directly from Equation (5). Particularly, after the direct Laplace transform, that equation considering the boundary mentioned above condition takes the following form:

(14) $$0=-U_{0}s^{\beta }C_{\infty }\left(s\right)+K\left[\frac{c_{s}}{s}-C_{\infty }\left(s\right)\right],$$

where $C_{\infty }\left(s\right)=L_{s}\left[c_{\infty }\left(z\right)\right]$—the Laplace transform with respect to longitudinal coordinate.

The following result can be derived from the last equation:

(15) $$C_{\infty }\left(s\right)=\frac{Kc_{s}}{s\left(U_{0}s^{\beta }+K\right)}=c_{s}\left(\frac{1}{s}-\frac{s^{\beta -1}}{s^{\beta }+\frac{K}{U_{0}}}\right).$$

After the inverse Laplace transform with respect to coordinate, the stationary solution $c_{\infty }\left(z\right)=L_{s}^{-1}\left[C_{\infty }\left(s\right)\right]$ takes the same form as Equation (13). This fact finally proves the reliability of the general solution (8).

For the case of insufficient convection (*z* → ∞), Equation (4) can be rewritten as follows:

(16) $$\frac{\partial^{\alpha }c_{0}\left(t\right)}{\partial t^{\alpha }}=K\left[c_{s}-c_{0}\left(t\right)\right],$$

where $c_{0}\left(t\right)=\underset{z\to \infty }{\lim}c\left(z,t\right)$—limiting concentration change in time.

After the direct Laplace transform, this equation considering the boundary condition mentioned above, takes the following form:

(17) $$\tau^{\alpha }C_{0}\left(\tau \right)=K\left[\frac{c_{s}}{\tau }-C_{0}\left(\tau \right)\right],$$

where $C_{0}\left(\tau \right)=L_{\tau }\left[c_{0}\left(t\right)\right]$—the Laplace transform with respect to time.

The following result can be derived from the last equation:

(18) $$C_{0}\left(s\right)=\frac{Kc_{s}}{\tau \left(\tau^{\alpha }+K\right)}=c_{s}\left(\frac{1}{\tau }-\frac{\tau^{\alpha -1}}{\tau^{\alpha }+K}\right).$$

After the inverse Laplace transform with respect to coordinate, the stationary solution $c_{0}\left(t\right)=L_{\tau }^{-1}\left[C_{0}\left(\tau \right)\right]$ takes the following form:

(19) $$c_{0}\left(t\right)=c_{s}\left[1-E_{\alpha ,1}\left(-Kt\right)\right].$$

This solution is a partial case of the general solution (7) reduced by the substitution of *z* → ∞, and $c_{0}\left(t\right)=\underset{z\to \infty }{\lim}c\left(z,t\right)$. This fact finally proves the reliability of the general solution (7).

## 3. Results

### 3.1. Parameter Identification of the Fractional-Order Model

For practical purposes, Equation (4) needs to evaluate five parameters: the limiting concentration *c_s_*, fractional orders *α* and *β*, as well as a convective ratio $U_{0}$ and mass transfer coefficient *K*. The following algorithm for their identification is proposed. Firstly, limiting concentration *c_s_* is evaluated under the saturation condition $c_{s}=\underset{z\to \infty }{\lim}c_{\infty }\left(z\right)$.

Secondly, Equation (17) is rewritten in a dimensionless form (Figure 2):

(20) $${\bar{c}}_{0}\left(t\right)=1-E_{\alpha ,1}\left(-Kt\right),$$

where ${\bar{c}}_{0}\left(t\right)=\frac{c_{0}\left(t\right)}{c_{s}}$—dimensional time-varying concentration.

Fractional order *α* and mass transfer coefficient *K* are evaluated based on the best fit of this solution with an available dataset of experimental parameters ${\bar{c}}_{0}^{<k>}$ for the *k*-th time moment *t_k_* ($k=\bar{1,N}$, where *N* is the total number of the time-varying dataset). For this purpose, the following least square error function should be minimized:

(21) $$R_{1}\left(\alpha ,U_{0}\right)=\sum_{k=1}^{N}{\left[1-E_{\alpha ,1}\left(-Kt_{k}\right)-{\bar{c}}_{0}^{<k>}\right]}^{2}\to min.$$

Such minimization is based on the following necessary and sufficient conditions:

(22) $$\left\{\begin{array}{c} \frac{\partial R_{1}}{\partial \alpha }=-\sum_{k=1}^{N}\left\{\left[1-E_{\alpha ,1}\left(-Kt_{k}\right)-{\bar{c}}_{0}^{<k>}\right]\frac{\partial E_{\alpha ,1}\left(-Kt_{k}\right)}{\partial \alpha }\right\}=0;\\ \frac{\partial R_{1}}{\partial K}=-\sum_{k=1}^{N}\left\{\left[1-E_{\alpha ,1}\left(-Kt_{k}\right)-{\bar{c}}_{0}^{<k>}\right]\frac{\partial E_{\alpha ,1}\left(-Kt_{k}\right)}{\partial K}\right\}=0. \end{array}\right.$$

After equal transformations and considering the first Expression (9), the following system of nonlinear equations can be obtained:

(23) $$\left\{\begin{array}{c} \sum_{k=1}^{N}\left\{\left[1-\sum_{i=0}^{\infty }\frac{{\left(-Kt_{k}\right)}^{i}}{\Gamma \left(\alpha i+1\right)}-{\bar{c}}_{0}^{<k>}\right]\sum_{i=1}^{\infty }i{\left(-Kt_{k}\right)}^{i}\frac{\Psi \left(\alpha i+1\right)}{\Gamma \left(\alpha i+1\right)}\right\}=0;\\ \sum_{k=1}^{N}\left\{\left[1-\sum_{i=0}^{\infty }\frac{{\left(-Kt_{k}\right)}^{i}}{\Gamma \left(\alpha i+1\right)}-{\bar{c}}_{0}^{<k>}\right]\sum_{i=1}^{\infty }\frac{i{\left(-Kt_{k}\right)}^{i}}{\Gamma \left(\alpha i+1\right)}\right\}=0. \end{array}\right.$$

where $\Psi \left(\theta \right)=\frac{d}{d\theta }\left\{ln\left[\Gamma \left(\theta \right)\right]\right\}$—the specialized function as a derivative of the natural logarithm of Euler’s gamma function.

Equation (23) allows for identifying both the parameters *α* and *K*.

Next, the evaluated parameter *K* is substituted for Equation (13), which can be rewritten in the following dimensionless form:

(24) $${\bar{c}}_{\infty }\left(z\right)=1-E_{\beta ,1}\left(-\frac{K}{U_{0}}z\right),$$

where ${\bar{c}}_{\infty }\left(z\right)=\frac{c_{\infty }\left(z\right)}{c_{s}}$—dimensional concentration for the stationary mass transfer process.

Fractional order *β* and convective ratio $U_{0}$ are evaluated based on the best fit of this stationary solution with an available dataset of experimental parameters ${\bar{c}}_{\infty }^{<j>}$ for the *j*-th number of the coordinate *z_j_* ($j=\bar{1,n}$, where *n* is the total number of the dataset). For this purpose, the following least square error function should be minimized:

(25) $$R_{2}\left(\beta ,\gamma \right)=\sum_{j=1}^{n}{\left[1-E_{\beta ,1}\left(-\frac{K}{U_{0}}z_{j}\right)-{\bar{c}}_{\infty }^{<j>}\right]}^{2}\to min.$$

Such minimization is based on the following necessary and sufficient conditions:

(26) $$\left\{\begin{array}{c} \frac{\partial R_{2}}{\partial \beta }=-\sum_{j=1}^{n}\left\{\left[1-E_{\beta ,1}\left(-\frac{K}{U_{0}}z_{j}\right)-{\bar{c}}_{\infty }^{<j>}\right]\frac{\partial E_{\beta ,1}\left(-\frac{K}{U_{0}}z_{j}\right)}{\partial \beta }\right\}=0;\\ \frac{\partial R_{2}}{\partial U_{0}}=-\sum_{j=1}^{n}\left\{\left[1-\sum_{i=0}^{\infty }\frac{{\left(-\frac{K}{U_{0}}z_{j}\right)}^{i}}{\Gamma \left(\beta i+1\right)}-{\bar{c}}_{\infty }^{<j>}\right]\frac{\partial E_{\beta ,1}\left(-\frac{K}{U_{0}}z_{j}\right)}{\partial U_{0}}\right\}=0. \end{array}\right.$$

After equal transformations and considering the second Expression (8), the following system of nonlinear equations can be obtained:

(27) $$\left\{\begin{array}{c} \sum_{j=1}^{n}\left\{\left[1-\sum_{i=0}^{\infty }\frac{{\left(-\frac{K}{U_{0}}z_{j}\right)}^{i}}{\Gamma \left(\beta i+1\right)}-{\bar{c}}_{\infty }^{<j>}\right]\sum_{i=1}^{\infty }i{\left(-\frac{K}{U_{0}}z_{j}\right)}^{i}\frac{\Psi \left(\beta i+1\right)}{\Gamma \left(\beta i+1\right)}\right\}=0;\\ \sum_{j=1}^{n}\left\{\left[1-\sum_{i=0}^{\infty }\frac{{\left(-\frac{K}{U_{0}}z_{j}\right)}^{i}}{\Gamma \left(\beta i+1\right)}-{\bar{c}}_{\infty }^{<j>}\right]\sum_{i=1}^{\infty }\frac{i{\left(-\frac{K}{U_{0}}z_{j}\right)}^{i}}{\Gamma \left(\beta i+1\right)}\right\}=0. \end{array}\right.$$

This system allows for identifying both the parameters *β* and $U_{0}$.

Therefore, parameter identification of the main parameters (*α*, *K*, *β*, and $U_{0}$) of the fractional convective mass transfer model in a porous medium is based on solving Equations (22) and (26). However, due to the complexity of this approach, it should be generalized for both cases as proposed below.

### 3.2. The Generalized Parameter Identification Approach

**Figure 3:**
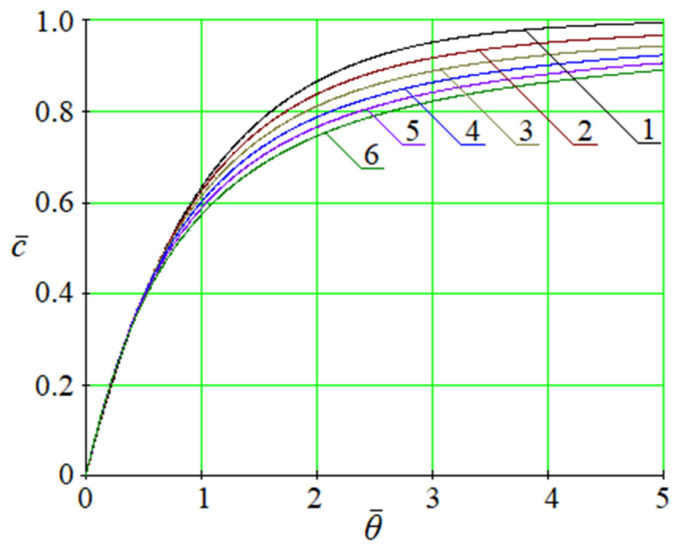
The dimensionless saturation curves (28) in terms of the dimensionless independent variable $\bar{\theta }=\kappa \theta$ for different numbers *γ*: 1—1.0; 2—0.9; 3—0.8; 4—0.7; 5—0.6; 6—0.5.

Despite the difference between these expressions, there is a way to present them similarly (Figure 3). For this purpose, the following single representation of solutions (20) and (24) is used:

(28) $$\bar{c}\left(\theta \right)=1-E_{\gamma ,1}\left(-\kappa \theta \right),$$

where *κ*—generalized coefficient; *γ*—index.

If coefficient *θ* = *K*, index *γ* = *α*, and independent variable *θ* = *t*, this solution corresponds to (20). Otherwise, in the case of $\theta =\frac{K}{U_{0}}$, *γ* = *β*, and *θ* = *z*, corresponding to (24).

Therefore, generally, the parameter identification of the fractional convective mass transfer model in a porous medium is based on the minimization of the following least square error:

(29) $$R\left(\gamma ,\kappa \right)=\sum_{j=1}^{n}{\left[1-E_{\gamma ,1}\left(-\kappa \theta_{j}\right)-{\bar{c}}_{j}\right]}^{2}\to min,$$

where *θ_j_*, ${\bar{c}}_{j}$—experimental datasets for independent parameters and the dimensionless concentration, respectively.

Such minimization is based on the following necessary and sufficient conditions:

(30) $$\left\{\begin{array}{c} \frac{\partial R}{\partial \gamma }=-\sum_{j=1}^{n}\left\{\left[1-E_{\gamma ,1}\left(-{\kappa \theta }_{j}\right)-{\bar{c}}_{j}\right]\frac{\partial E_{\gamma ,1}\left(-{\kappa \theta }_{j}\right)}{\partial \gamma }\right\}=0;\\ \frac{\partial R}{\partial \kappa }=-\sum_{j=1}^{n}\left\{\left[1-E_{\gamma ,1}\left(-{\kappa \theta }_{j}\right)-{\bar{c}}_{j}\right]\frac{\partial E_{\gamma ,1}\left(-{\kappa \theta }_{j}\right)}{\partial \kappa }\right\}=0, \end{array}\right.$$

or in the following form:

(31) $$\left\{\begin{array}{c} \sum_{j=1}^{n}\left\{\left[1-\sum_{i=0}^{\infty }\frac{{\left(-\kappa \theta_{j}\right)}^{i}}{\Gamma \left(\gamma i+1\right)}-{\bar{c}}_{j}\right]\frac{\partial \sum_{i=0}^{\infty }\frac{{\left(-\kappa \theta_{j}\right)}^{i}}{\Gamma \left(\gamma i+1\right)}}{\partial \gamma }\right\}=0;\\ \sum_{j=1}^{n}\left\{\left[1-\sum_{i=0}^{\infty }\frac{{\left(-\kappa \theta_{j}\right)}^{i}}{\Gamma \left(\gamma i+1\right)}-{\bar{c}}_{j}\right]\frac{\partial \sum_{i=0}^{\infty }\frac{{\left(-\kappa \theta_{j}\right)}^{i}}{\Gamma \left(\gamma i+1\right)}}{\partial \kappa }\right\}=0. \end{array}\right.$$

After considering the following derivatives from the Mittag-Leffler function:

(32) $$\frac{\partial \sum_{i=0}^{\infty }\frac{{\left(-\kappa \theta_{j}\right)}^{i}}{\Gamma \left(\gamma i+1\right)}}{\partial \gamma }=-\sum_{i=0}^{\infty }{\left(-\kappa \theta_{j}\right)}^{i}\frac{\Psi \left(\gamma i+1\right)}{\Gamma \left(\gamma i+1\right)};\frac{\partial \sum_{i=0}^{\infty }\frac{{\left(-\kappa \theta_{j}\right)}^{i}}{\Gamma \left(\gamma i+1\right)}}{\partial \kappa }=\sum_{i=1}^{\infty }\frac{i{\left(-\kappa \theta_{j}\right)}^{i}}{\kappa \Gamma \left(\gamma i+1\right)},$$

the system of the nonlinear Equation (31) can be rewritten like (23) and (27):

(33) $$\left\{\begin{array}{c} \sum_{j=1}^{n}\left\{\left[1-\sum_{i=0}^{\infty }\frac{{\left(-\kappa \theta_{j}\right)}^{i}}{\Gamma \left(\gamma i+1\right)}-{\bar{c}}_{j}\right]\sum_{i=1}^{\infty }{i\left(-\kappa \theta_{j}\right)}^{i}\frac{\Psi \left(\gamma i+1\right)}{\Gamma \left(\gamma i+1\right)}\right\}=0;\\ \sum_{j=1}^{n}\left\{\left[1-\sum_{i=0}^{\infty }\frac{{\left(-\kappa \theta_{j}\right)}^{i}}{\Gamma \left(\gamma i+1\right)}-{\bar{c}}_{j}\right]\sum_{i=1}^{\infty }\frac{i{\left(-\kappa \theta_{j}\right)}^{i}}{\Gamma \left(\gamma i+1\right)}\right\}=0. \end{array}\right.$$

This system of equations allows for identifying both the parameters *γ* and *κ*.

Therefore, the generalized scientific and methodological approach for evaluating the main parameters of the fractional convective mass transfer model in a porous medium is based on solving the generalized system of the nonlinear Equation (33).

### 3.3. The Evaluation Algorithm

The significance of such an approach is associated with the following algorithm of its realization. The initial value *γ*_0_ = 1 of the parameter *γ* is chosen in the initial step. Then, the first approximation *κ*_1_ of the parameter *κ* is evaluated numerically from the second Formula (33). Finally, the first approximation *γ*_1_ of the parameter *γ* is evaluated numerically from the first Formula (33). This cycle should be realized during *p* ≥ 1 step till condition (29) is fulfilled.

A rhomb-shaped pneumatic classifier was considered to obtain the evaluation dataset. Such an apparatus allows for effectively separating samples of granular materials (porous ammonium nitrate NH_4_NO_3_, superphosphate Ca(H_2_PO_4_)_2_·H_2_O, and carbamide CO(NH_2_)_2_), reaching relatively high values of the target fraction.

**Figure 4:**
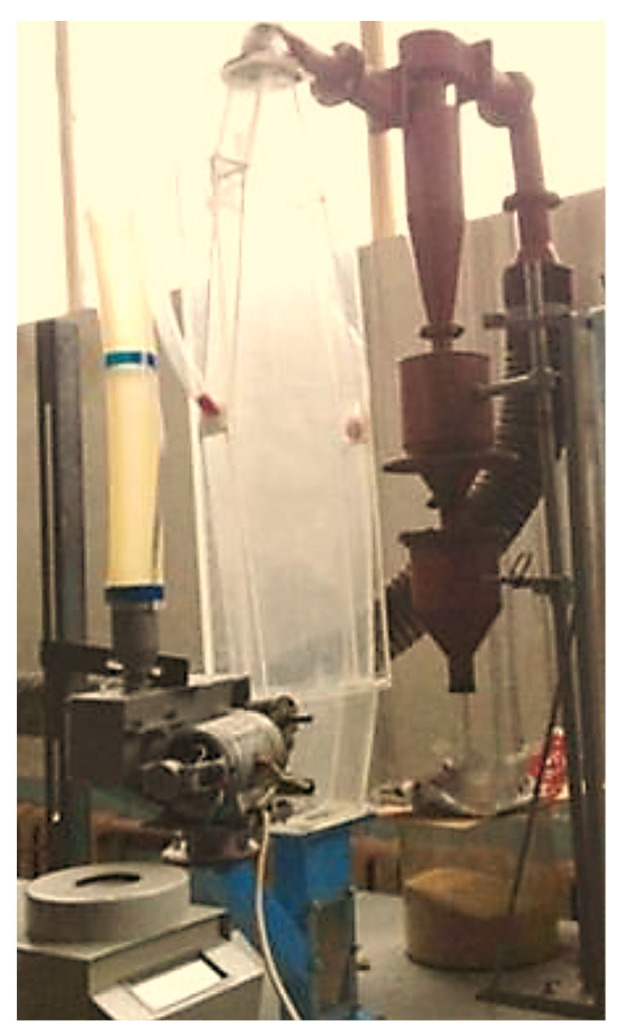
The experimental setup.

The experimental setup is presented in Figure 4. It consists of a pneumatic classifier, cyclone, gas blower for creating a suction flow, regulating valve, and separate tanks for collecting coarse and fine fractions.

Sieves of various sizes are used to obtain the different charges. The Digimizer 6.3.0 software can be additionally applied to decrease the root mean square deviation up to 0.014 mm.

The pressure and the airflow in the apparatus at different cross-sections were measured by the U-shaped liquid pressure gauges and the Pitot–Prandtl tube (Sumy, Ukraine), respectively. A sloping tube with a micromanometer MMN-240(5)-0.1 (Poznan, Poland) allowed for controlling the pressure difference at the cross-sections.

The experimental data (concentration for the considered case study) were determined by weighting batches of the porous granular material using the electronic scale Momert-6000 (Bratislava, Slovakia) with an accuracy of 0.1 g.

The dataset with the total number of the generated experimental *n* = 30 was considered for the case study of the pneumatic classification of the porous granular material.

The evaluation results are summarized in Table 1.

**Figure 5:**
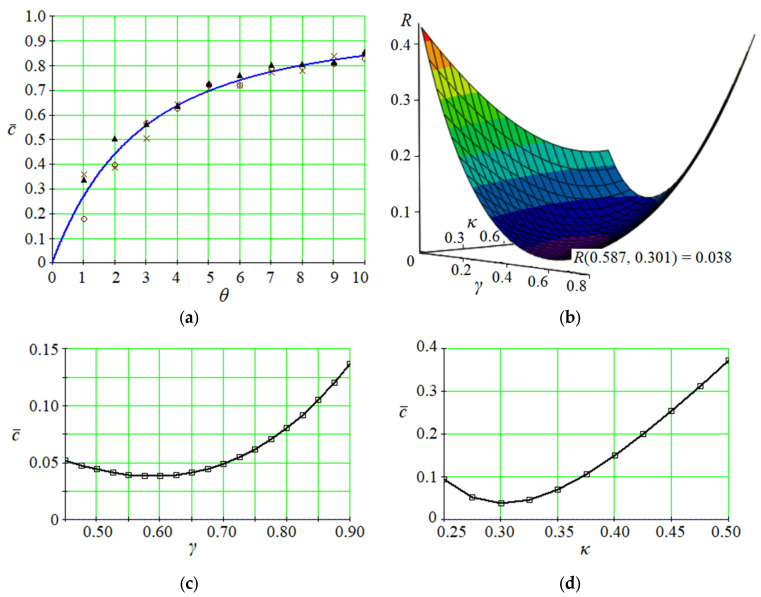
Parameter identification results: (**a**)—approximation of the evaluation dataset (circle: 1–10; cross: 11–20; triangle: 21–30); (**b**)—the least square error surface; (**c**,**d**)—plane curves of the least square error minimization by parameters *γ* and *κ*, respectively.

The dataset and the corresponding parameter identification results are presented in Figure 5.

The following parameters have been identified after *p* = 9 approximation cycles: *γ* = 0.587 and *κ* = 0.301. In this case, the minimized least square error (28) equals 0.038.

Overall, the reliability of the proposed procedure is proven by small values of the averaged relative error between the initial and evaluated datasets of concentrations. Particularly, the average relative error is equal to 2.1%.

## 4. Discussion

The obtained results significantly extend previous studies in the mathematical modeling of working processes in mass transfer equipment. Mainly, the mathematical model of the concentration changes [33] can been improved in considering the proposed model’s fractional origin. This also makes it possible to improve practical ways for ensuring the reliability of the linear fractional advection–diffusion, advection–dispersion, and convection–dispersion models [35], i.e., through heteroporous membranes [36,37]. It also allows for improving the pneumatic classification process for granular material in rhomb-shaped apparatuses [38], intensifying the cooling process during obtaining granular materials [30], decreasing dust emission by monodisperse system technology [39], and reducing the technogenic impact on the environment from the emissions of heat power engineering by using highly efficient equipment [40].

The research findings are also helpful in solving numerous practical problems with mass transfer equipment. Particularly, the obtained solution allows for identifying parameters of the fractional-order mathematical model of particle sedimentation [41], convective heat transfer in porous insulation layers [9], and hydrodynamics of viscoelastic fluid flows [22]. They are also applicable in practice to solve the coupled problem of heat and mass transfer with non-stationary boundaries for the consequent modeling of the oscillation’s propagation in a working medium of a vibrational priller.

Moreover, the applied model can be practically applied to extend the application of a functionally graded fractional porous medium in biomechanical applications [42].

The practical significance of the obtained results corresponds to the significance of the convective mass transfer model in terms of theoretical substantiation of mathematical models of oil filtration through a porous medium [27]. It also allows the implementation of the developed model to design inertial-filtering separators [29] with the consequent evaluation of their operating parameters.

The theoretical value of the research is in substantiating the scientific fundamentals of evaluating orders of fractional derivatives of the mathematical model for convective mass transfer. The developed parameter identification approach will allow for evaluating the porosity and fractional dimension of porous materials based on experimental data.

The limitations of the study are mainly predetermined by the case of a predominantly convective mode for significant values of the multiplication of the Reynolds and Prandtl numbers. However, further extending the proposed model to diffusion and Taylor dispersion in higher space dimensions using, e.g., Riesz–Feller fractional operators [43], will allow for eliminating this limitation.

More precise studies of the impact of operating parameters on the size of pores, as well as the physical and chemical properties of the resulting granules, will also be carried out further by using an electron microscope.

## 5. Conclusions

Thus, a fractional mathematical model of the convective mass transfer has been developed. It extends the available models and makes the fundamentals in considering new phenomena in hydromechanics and heat and mass transfer, considering the fractal nature of the porous medium.

During the study, the fractional convective mass transfer equation was solved analytically based on the Laplace transform with respect to time and coordinate and further representation using the Mittag-Leffler function and Duhamel integral.

The obtained generalized solution has been justified by four available partial solutions (i.e., integer parameters, stationary mode, saturation mode, and without significant convection).

The experimental results have been realized for the case of a hydrodynamic classifier for porous granular materials. They have been approximated by analytical curves. Such an approach has allowed for identifying parameters of the proposed fractional-order mathematical model.

As a result, the stationary and non-stationary convective mass transfer models in a porous medium have been solved. The corresponding parameter identification approach has been developed. Moreover, the impact of the fractional orders for a porous medium on the behavior of spatial surface and plan curves has been presented graphically for the dimensionless concentration. The reliability of the proposed approach has been proven by the average error of the approximation equal to 2.1%.

Overall, a scientific and methodological approach to parameter identification of the fractional convective mass transfer model has been developed. It has allowed for evaluating the parameters of this model by an experimental dataset of concentration changes during pneumoclassification of porous granular materials. The corresponding algorithm of such a parameter identification has been described. The evaluation example has proved the developed approach with the least square error of 0.038.

The scientific novelty of the research is in considering the fractional-order nature of the mathematical model for convective mass transfer, which has created the scientific foundation of further substantiating the anomalous hydromechanical advection–dispersion in porous structures of fractal nature.

The main results of the research help solve practical problems in chemical process engineering (hydrodynamic classification of porous granular and grain materials, vibrational prilling, convective cooling of porous fertilizers, and heat and mass transfer in porous nozzles). They can also be valuable in biomechanical engineering (e.g., studying the processes in a functionally graded fractional porous medium in human meniscus, intensifying particle sedimentation in elastic membranes, and so on).

## Figures and Tables

**Table 1:** The evaluation results data.

Initial Data										
*θ_j_*	1	2	3	4	5	6	7	8	9	10
${\bar{c}}_{j}$	0.177	0.397	0.566	0.624	0.721	0.720	0.787	0.782	0.805	0.826
*θ_j_*	11	12	13	14	15	16	17	18	19	20
${\bar{c}}_{j}$	0.357	0.387	0.502	0.641	0.704	0.719	0.772	0.779	0.835	0.841
*θ_j_*	21	22	23	24	25	26	27	28	29	30
${\bar{c}}_{j}$	0.337	0.504	0.561	0.634	0.724	0.759	0.803	0.807	0.812	0.854
Average Data										
*θ_j_*	1, 11, 21	2, 12, 22	3, 13, 23	4, 14, 24	5, 15, 25	6, 16, 26	7, 17, 27	8, 18, 28	9, 19, 29	10, 20, 30
${\bar{c}}_{j}$	0.290	0.429	0.543	0.633	0.716	0.733	0.787	0.789	0.817	0.840
Evaluated Data										
$\bar{c}\left(\theta_{j}\right)$	0.268	0.440	0.556	0.637	0.695	0.739	0.773	0.799	0.821	0.838
Relative Deviation										
$\frac{\left|\bar{c}\left(\theta_{j}\right)-{\bar{c}}_{j}\right|}{{\bar{c}}_{j}}$	0.082	0.025	0.023	0.006	0.030	0.009	0.019	0.013	0.004	0.003

## Data Availability

Data is available on request from the authors.

## Nomenclature

*C*, *c*	mass concentration	kg/kg
$\bar{c}$	dimensionless concentration	
*c_s_* $,\;c_{\infty }$	limiting concentrations	kg/kg
*D*_0_	diffusion coefficient	m^2^/s
*E*	Mittag-Leffler function	
*Fo*	Fourier number	
*H*	Heaviside function	
*i*, *j*, *k*	sequence numbers	
*K*	mass transfer coefficient	kg/s
ℒ	Laplace transform sign	
*N*, *n*	dataset number	
*p*	approximations number	
*Pr*	Prandtl number	
*R*	driving force	kg/s
*r*	radius-vector	m
*Re*	Reynolds number	
*R*_1_, *R*_2_	error functions	
*s*	derivative operator	
*t, τ*	time	s
*U*_0_	velocity	m/s
*z*	coordinate	m
*α*, *β*	fractional orders	
Γ	Euler’s gamma function	
*γ*	index	
*ζ*	coordinate parameter	m
*θ*	dimensionless argument	
*κ*	generalized coefficient	
Ψ	specialized function	
